# Palliative care patients’ attitudes and openness towards psilocybin-assisted psychotherapy for existential distress

**DOI:** 10.3389/fpsyt.2024.1301960

**Published:** 2024-04-18

**Authors:** Julia Ruixi Wang, Samuel J. Mendez Araque, Gina Micciche, Andrew McMillan, Emily Coughlin, Rosalie Mattiola, Diana English, Kristopher Kaliebe

**Affiliations:** ^1^ MD Program, Morsani College of Medicine, University of South Florida, Tampa, FL, United States; ^2^ Undergraduate Studies, College of Arts and Sciences, University of South Florida, Tampa, FL, United States; ^3^ Department of Medical Education, University of South Florida, Tampa, FL, United States; ^4^ Department of Obstetrics and Gynecology, University of South Florida, Tampa, FL, United States; ^5^ Department of Internal Medicine, University of South Florida, Tampa, FL, United States; ^6^ Department of Psychiatry, University of South Florida, Tampa, FL, United States

**Keywords:** psilocybin, psychedelic assisted psychotherapy, incurable illnesses, palliative care, existential distress, cancer

## Abstract

**Introduction:**

Patients with incurable illnesses often experience existential distress, profoundly impacting their well-being. Current medical approaches have limitations in addressing these burdens. Psilocybin, a promising psychedelic compound, may offer therapeutic benefits. This pilot survey study aimed to investigate the attitudes and openness toward psilocybin-assisted psychotherapy (PAT) among patients with incurable illnesses. The objective is to assess patients’ attitudes toward PAT and identify potential barriers and concerns, including exploring the association between beliefs in psilocybin’s therapeutic benefits and interest in receiving this treatment.

**Methods:**

The survey study was conducted at the Tampa General Hospital Palliative Care Outpatient office in the United States. Participants were 32 English-fluent patients, aged 18 or older, with incurable illnesses. The survey included demographic questions, a validated tool to measure existential distress, and questions about knowledge and concerns regarding psilocybin. Attitudes toward PAT and interest in its future use were assessed using Likert scale responses.

**Results:**

Among the 31 analyzed participants, 51.6% expressed interest in future psilocybin treatment, while 32.3% did not indicate interest. Belief in the psilocybin’s therapeutic benefits for stress and anxiety significantly correlated with interest in use. Concerns included risk of psychosis, lack of trained providers, and potential for exploitation. No demographic factors were associated with interest or levels of distress.

**Conclusions:**

This pilot study provides insights into the attitudes and concerns toward PAT among patients with incurable illnesses. Over half of participants expressed interest. However, concerns regarding its use were identified, with patients’ concern for the risk of exploitation associated with PAT as an especially novel concern documented in this patient population. This highlighted the need for further education of risks and benefits or PAT by trained clinicians and rigorous training of clinicians with the establishment of safeguards against exploitation. Further research is necessary to explore the potential benefits of PAT and related non-psilocybin psychedelic compounds in addressing existential distress among patients with incurable illnesses.

## Introduction

Diagnosis of a terminal or life-threatening illness profoundly alters individuals’ conception of their social, emotional, physical, and spiritual selves (1); the progression of the disease may induce distressing emotions, including death anxiety, despair, a sense of burden to others, loss of meaning, and hopelessness. Existential distress (EXD) is a psychological disturbance that arises when questioning life purpose, meaning, existence, or life after death, and is significantly associated with anxiety about end-of-life and depression (2). Patients in hospice and/or palliative care can grapple with psychological, emotional, social, and spiritual burdens related to EXD, profoundly impacting their quality of life (2–4).

Current medical approaches, such as psychotherapy alone or combined with pharmaceutical management, may not fully address the unique burden and distress faced by patients with incurable illnesses. These conventional methods necessitate extended periods to take full effect and can come with unwanted side effects, requiring trial and error to identify the most suitable treatment. For terminally ill pts with distress, depression, and/or anxiety, one study showed that half of patients did not achieve symptom remission with serotonin reuptake inhibitor medication (5).

Emotional and spiritual support from chaplains or counselors is an integral component of palliative care. Palliative care providers believe that existential distress is a common experience that is frequently insufficiently treated within the current treatment framework (6). Spiritual support from chaplains or counselors may be limited by availability, the diverse spiritual needs of patients, and the subjective nature of existential distress. EXD is a psycho-social and spiritual challenge that evades current medicalized approaches, and palliative care providers believe innovative approaches like psychedelic-assisted psychotherapies (PAT) are promising avenues to alleviate distress and improve quality of life (6).

Psilocybin, a psychedelic compound acting as a 5HT2A serotonin receptor agonist, induces hallucinations, euphoria, and a sense of unity, oneness, and peace, thereby reducing symptoms of distress (7). Clinical trials of single moderate-dose psilocybin treatment paired with psychotherapy have demonstrated rapid onset and sustained symptom reduction in anxiety and depression in patients with life-threatening cancer, with peak anxiety reduction occurring 1 to 3 months post-treatment (8, 9). The positive effects of PAT persisted for at least 4.5 years without additional psychedelic treatments (10). The rapid action with psilocybin treatments is particularly valuable for terminally ill patients. In patients with major depressive disorder, PAT led to substantial and enduring antidepressant effects, persisting for at least 4 weeks in 71% of participants (11). Psilocybin demonstrated a low potential for addiction and a minimal adverse event profile, suggesting therapeutic advantages with reduced associated risks (7). The effect sizes observed with psilocybin-assisted therapy surpass those reported with these traditional treatment approaches (11).

While psilocybin is one well-known psychedelic compound that has garnered significant attention in this context, it’s essential to recognize that it is just one piece of the broader landscape of non-psilocybin psychedelics, such as classic serotonergic psychedelic lysergic acid diethylamide (LSD) and atypical psychedelics such as 3,4-Methylenedioxymethamphetamine (MDMA). A 1973 study investigating the properties of LSD for patients with cancer and other serious illnesses showed promising results, with 70.9% of patients seeing an improvement in emotional and psychological distress, as measured by scales of depression, anxiety, psychological isolation, fear of death, and pain (12). A palliative care population receiving LSD indicated positive effects on existential and spiritual well-being, quality of life, acceptance, and with 60–80% of the patients showing significant reductions in depression and anxiety compared to baseline even after 4 years (13). In a pilot study, 20 patients with life-threatening illnesses received MDMA with two psychotherapy sessions resulted in a significant reduction in trait anxiety compared to placebo in patients with anxiety secondary to a life-threatening illness, with results remaining stable after 6 months and 1 year (14). Moreover, a recent survey study with 27 respondents has shown that co-use of 3,4-Methylenedioxy methamphetamine (MDMA) with psilocybin/LSD anecdotally reduces challenging experiences such as anxiety and fear, while enhancing positive experiences such as self-compassion and gratitude associated with psilocybin/LSD use (15). These survey results were limited by use of convenience sample and small sample size. The potential of combining psychedelic treatment and psychotherapy may not only mitigate the symptoms of, but to enhance the quality of life for individuals with incurable and terminal diagnoses (16, 17). With potential co-use with MDMA to buffer against aspects of challenging experiences and integration of the experience with psychotherapy, the growing body of research underscores the potential therapeutic value of psychedelic-assisted therapy, not just for individuals with life-threatening illnesses but also for those with various psychiatric conditions, thus offering new avenues to improve mental health outcomes.

The overall attitudes towards psychedelic psychotherapy has shifted towards more support and interest in the benefits of PAT. Studies centered around international cancer healthcare practitioners (18), Canadian adults when asked existential distress (19), and American psychiatrists (12) have shown interest in psychedelic psychotherapy for their respective patient populations. Healthcare providers for patients with cancer in New Zealand and America agreed that psychedelic-assisted therapy holds promise as a potential intervention for addressing the psychological and existential distress experienced by cancer patients (18). Importantly, cancer healthcare providers were more likely to theoretically refer PAT to patients if they had an incurable cancer as opposed to a potentially curative cancer, and practitioners were willing to refer their patients to clinical trials investigating psychedelic-assisted therapy, even during intensive curative treatment (18). In a recent study, 79.3% of Canadian adults showed strong support and high social acceptability for PAT for potential treatment of existential distress (19). Interestingly, factors positively associated with acceptability included previous psilocybin use, exposure to palliative care practices, and a progressive political orientation (19). In a 2023 repeat of a 2016 survey study of American psychiatrists, there was a profound positive shift in attitudes towards the therapeutic potential of psychedelics over the years, with 80.9% of respondents believing psychedelics showed promise in treating psychiatric conditions and 93.9% supporting research into their therapeutic potential for psychiatric conditions (12). Notably, half of respondents reporting intentions to incorporate psychedelic-assisted psychotherapy into their practice (12). This shows a large shift in the attitudes and perception of psychedelic psychotherapy, further necessitating the need to assess for patients’ acceptability and feasibility.

It is essential to assess the acceptability and feasibility of PAT within the terminally ill and palliative care population, as PAT could potentially address EXD and its related burdens with minimal side effects. Thus, the current survey study seeks to explore the attitudes, openness, and knowledge of psilocybin psychotherapy among patients with incurable illnesses and aims to identify any barriers to its implementation. In doing so, it contributes to the broader conversation surrounding the application of non-psilocybin psychedelics in the realm of palliative care, offering hope and relief to those facing life’s most challenging moments.

## Materials and methods

### Approval

This survey study was granted exemption from the Institution Review Board at the University of South Florida (USF) and was approved by the Office of Clinical Research at Tampa General Hospital (TGH) with the reference number STUDY004118.

### Design

This prospective cohort survey study utilized survey assessment to accurately measure individual attitudes and openness towards psilocybin psychotherapy.

### Data collection

The survey was hosted on USF’s Qualtrics platform, ensuring data privacy and confidentiality. No patient-identifying information was collected, and paper surveys were destroyed after being entered into Qualtrics. Survey data was entered without input from study staff, and anonymous responses were analyzed by USF researchers only. Participant confidentiality was protected by presenting aggregated results, and no identifying information was included in quotes in open-ended responses.

### Instruments and measures

The survey included demographic questions, health history, and validated tools to assess existential distress. The 10-question Existential Distress Scale (EDS) (2) was used to evaluate existential distress. Additionally, non-validated questions were included to measure knowledge and concerns about psychedelics.

### Setting and participants

This survey was administered in person by two trained research team members at the TGH Palliative Care Outpatient office from July 29, 2022 to August 29, 2022. Convenience sampling was used by clinic staff to identify participants who were physically and cognitively able to provide informed consent, were at least 18 years of age, fluent in English, and had a diagnosis of terminal or incurable illness. Patients were excluded if they were undergoing an acute crisis or failed the English fluency test.

Verbal consent was obtained prior to initiating the questionnaire, and patients were given the option to complete the survey on paper or on an iPad with verbal assistance from the research team. All patients chose to complete the paper surveys with verbal assistance. Survey administrators were trained to maintain neutrality and instructed to ask questions verbatim. Field notes were taken for survey study improvement. At the conclusion of the survey study, participants were provided with a list of psychological and spiritual support resources, and national psilocybin-therapy programs.

### Statistical methods

Responses to EDS questions were compiled and summed to create a total EDS score. Total EDS scores were compared by demographic and other key variables using nonparametric Mann-Whitney U and Spearman’s rho tests. Participants’ interest in future use of psilocybin was collected using a four-point Likert scale which was then recoded to two levels of “agree” and “disagree”. Interest in psilocybin was assessed with other categorical variables using chi-square and Fisher’s exact tests. All tests used a significance level of p<0.05 and exact test significances are reported. Analysis was completed using SPSS Version 27.

## Results

Out of 47 considered patients, 5 were not approached due to acute distress, and 10 declined consent. Among the remaining patients, 32 took the survey, with one incomplete response. No patient submitted more than one survey. The most common reason for refusing the survey was time constraints.

A total of 31 participants were analyzed. Patients were 51.6% female with an average age of 51.39 ± 13.32. Participants were Caucasian 16 (51.6%), African American 7 (22.6%), mixed race 4 (12.9%), or other race 3 (9.7%). When asked if they would be interested in receiving psilocybin as a potential future treatment, 16 (51.6%) agreed that they would, while 10 (32.3%) disagreed, and 5 (16.1%) did not answer. Within those who agreed, 7 (22.6%) strongly agreed, and within those who disagreed, 8 (25.8%) strongly disagreed. Interest in psilocybin as a potential future treatment was not associated with any demographic factors including gender (p=0.701), race (p=1.000), ethnicity (p=0.626), income (p=0.601), or age (p=0.623). Belief that psilocybin shows promise in treating stress or anxiety was significantly associated with interest in psilocybin as a treatment (p=0.005). Interest in psilocybin as a future treatment was also associated with agreement that psilocybin should be legally accessible for medical use (p=0.003), spiritual/religious use (p=0.002) and recreational use (p=0.021).

Interest in psilocybin use was not significantly correlated with prior experience taking a hallucinogen, medical cannabis, or recreational cannabis (p=0.66). Hallucinogen was defined in the survey as “LSD, psilocybin mushroom, mescaline, peyote, Ayahuasca, MDMA, etc.” 13 (41.9%) of respondents indicated they have taken a hallucinogen, with most respondents answering yes ranging in age from 26 to 64, with a median age of 56. Respondents indicating yes were mostly white or European, with only one respondent identifying as African. Similarly, subjects were varied in religiosity, with Christianity being the most represented group with 6 yes responses, followed by spiritual with 3, agnostic with 2, and pagan with 1. One participant declined to answer. Respondents were also varied in educational attainment, with the highest degree being a bachelors achieved by two respondents. 16 (51.6%) of respondents indicated they have taken cannabis or “marijuana” for medical reasons. Of these respondents, 8 have also previously tried hallucinogens. Their ages ranged from 20 to 62, with a median age of 45. These respondents were more varied demographically, with 5 respondents identifying as African. Notably, only 2 respondents identifying as African reported not having used medical cannabis. Those who have tried medical cannabis were more likely to be varied spiritually, with 5 respondents being Christian, 5 being spiritual but not religious, 4 being agnostic or atheist, 1 respondent answering wicca, and 1 preferring not to answer. 14 (45.2%) indicated they have taken cannabis for recreational use. These respondents range from 26 to 67, with a median age of 48. These respondents were more likely to be white or European, representing 8 of the surveyed. Of this group there was one Indian respondent. Educational achievement was variable in this group, ranging from some high school to one respondent with a bachelor’s degree. Additionally, religious beliefs were varied in this group, representing 6 Christians, 4 spiritual but not religious people, 2 atheists, 1 wiccan, and 1 pagan. 11 (35.5%) of respondents preferred not to answer whether they have taken cannabis recreationally. 20 (64.5%) of participants report history of taking a prescribed medication for depression, anxiety, or other psychological symptoms (i.e. antidepressants, anti-anxiety medication). This group was the most varied without discernable mode of religion, race, or educational achievement. The ages of these respondents had the largest range from 20 to 76. The median age was 50. 14 (46.7%) of participants responded that they have witnessed someone under the influence of psilocybin in a recreational setting. Of those who have, 7 (50%) described the experience as primarily or somewhat positive. 5 (35.7%) participants reported describing the experience as neutral. 2 (14.2%) participants described the observed situation as primarily negative.

Average total EDS score was 16.13 ± 8.19, ranging from 10-38 out of 50 with 50 being unbearably distressed for each item. A total of 10 (33.3%) respondents reported either “great” or “unbearable” distress on at least one scale item. The item with the highest reported distress was “How distressed have you been by the thought that you are or will be a burden to others”, with 20% reporting great or unbearable distress for this item. The item with the least distress was “ How distressed have you been by the thought that you have nothing to live for” with zero participants reporting great or unbearable distress, and 86.7% reporting no distress for this item. In **Table 1**, total EDS scores were not associated with any demographic factors including gender (p=0.142), race (p= 0.992), ethnicity (p=0.448) or age (Spearman’s rho correlation -0.022, p=0.906). Additionally shown in **Table 2**, EDS scores were not associated with interest in psilocybin as a future treatment (p=0.507). EDS score did not differ by the factor “Have you ever taken a hallucinogen” (p=0.662).

**Table 1:** EDS scores and patient characteristics.

Variable		EDS Score (mean ± SD)	p-value
Gender			.142
	Male	19.00 ± 10.30	
	Female	13.63 ± 4.84	
Race			.992
	White	14.93 ± 6.27	
	Black/African American	20.71 ± 12.93	
	Mixed Race	15.75 ± 7.14	
	Other	13.67 ± 8.28	
Hispanic ethnicity			.448
	Hispanic	12.80 ± 3.27	
	Non-Hispanic	17.00 ± 8.88	
I would be interested in receiving psilocybin as a potential future treatment			.057
	Disagree	11.78 ± 1.92	
	Agree	18.81 ± 9.91	
Have you ever taken a hallucinogen			.662
	No	15.33 ± 7.18	
	Yes	17.33 ± 9.73	

**Table 2:** Hypothesis testing of existential distress score with survey questions.

Question Surveyed	T-Test Score
Interest in Receiving Psilocybin Therapy in the Future	0.057
Have You Ever Taken a Hallucinogen	0.205
I Have Been Well Supported in my Spiritual and Emotional Care	0.826
*The significance level for all tests is 0.050*	

**Figure 1:**
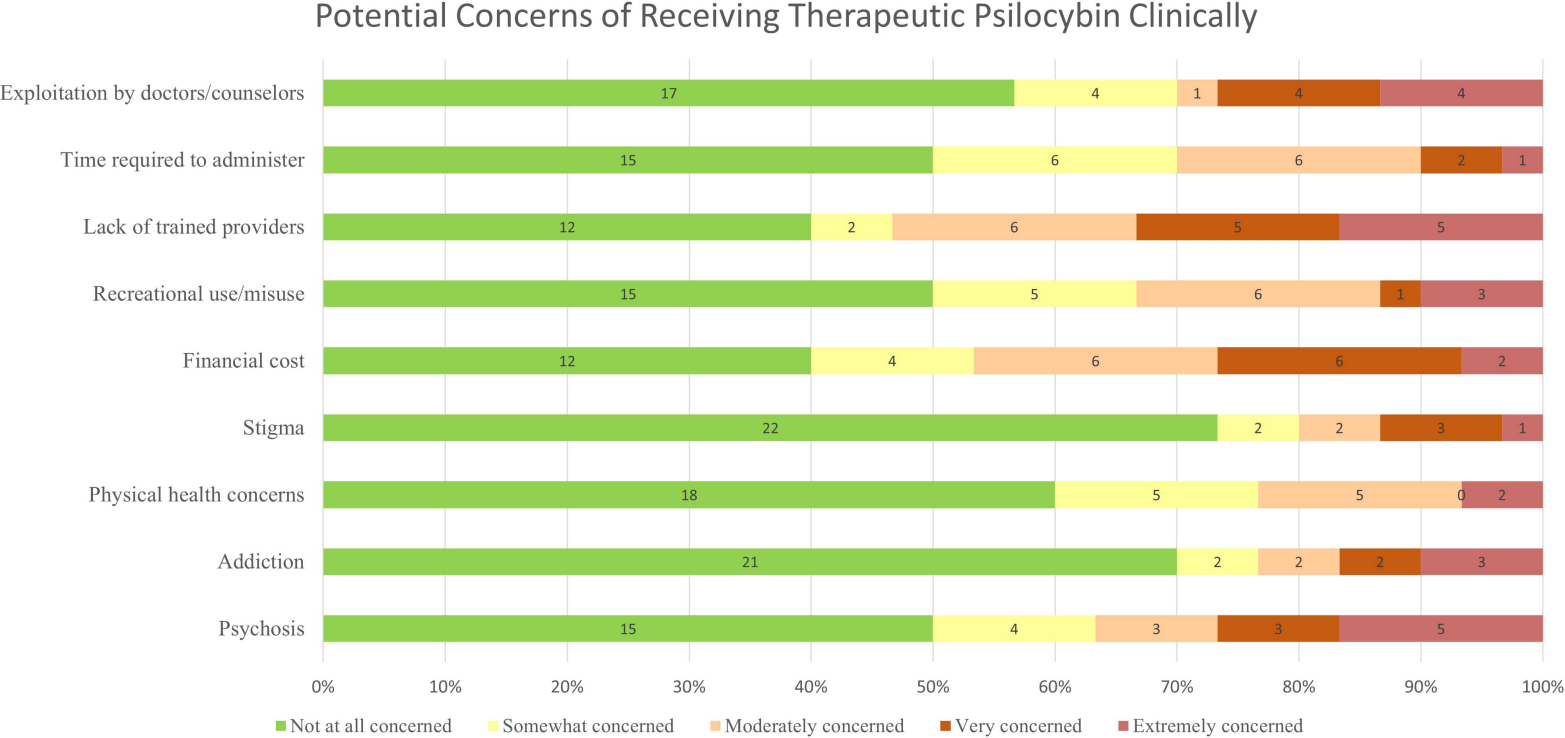
Reported concerns regarding PAT.

When asked about concerns of receiving therapeutic psilocybin in a clinical setting, participants reported being “extremely concerned” regarding psychosis (16.1%), lack of trained providers (16.1%), and exploitation by doctors/counsellors (12.9%). Participants reported being “not at all concerned” regarding stigma (71.0%), and addiction (67.7%). Responses regarding concerns are reported in **Figure 1**. No individual concerns were significantly associated with interest in psilocybin as a future treatment.

**Figure 2:**
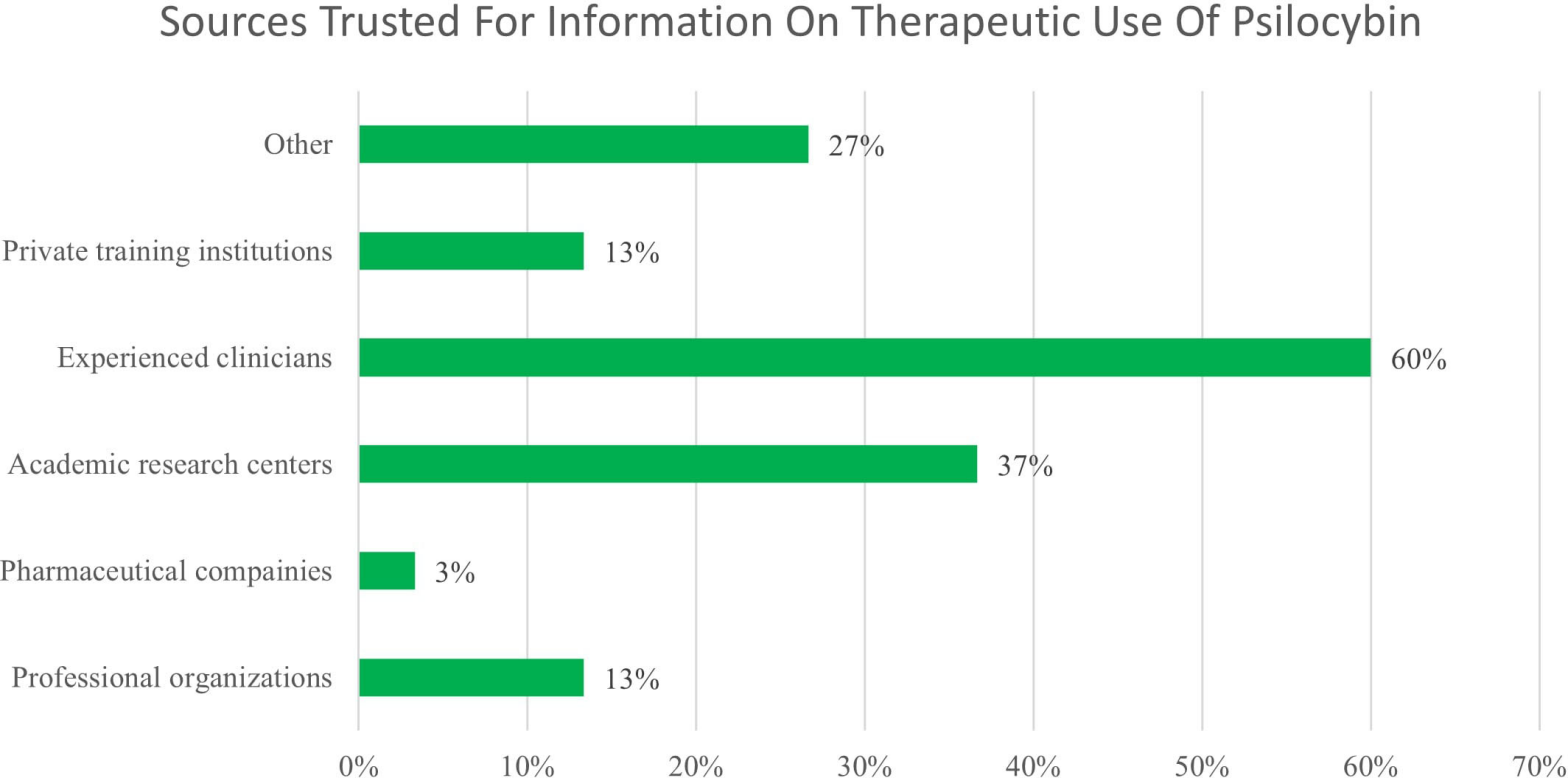
Reported sources of trust regarding information.

Only 9.7% of participants stated that they strongly agree with the statement “I have good knowledge of the potential therapeutic uses of psilocybin” and 16.1% strongly agree with “I have good knowledge of the risks and side effects of psilocybin.” Participants were asked where most of their knowledge on psilocybin has come from and could select all options that applied. The most commonly selected options were personal experience (33%), informal conversations (27%), and movies/TV (20%). When asked what sources they would trust for information, participants selected experienced clinicians (60%), academic research centers (37%), other (27%), private training institutions (13%), and pharmaceutical companies (3%), shown in **Figure 2**. Participants who reported “other” said they would trust the Food and Drug Administration, other’s personal experiences, or that they do not trust any of these sources (12.9%). Participants were asked to select all that apply for the prompt “Current data indicate psilocybin may be useful for treating…”. Most participants (50%) selected “other” and wrote in “I don’t know”. Among the options given, the most common selections were anxiety disorders (46%), depressive disorders (38%), personality disorders (35%), and palliative care (31%). No participants selected “none of these” and only 4% selected “cancer and overall wellness”.

## Discussion

The present survey study aimed to explore the attitudes and perceptions of a group of 31 participants regarding psilocybin as a potential future treatment. Interestingly, over half of the participants (51.6%) expressed interest in receiving PAT, while 32.3% disagreed. Notably, a significant portion of participants strongly agreed (22.6%) or strongly disagreed (25.8%), indicating a clear polarization in opinions within the sample. No demographic factors were found to be associated with interest in psilocybin as a future treatment. However, this may be due to small sample size and lack of power in this survey study.

### EXD and psilocybin

The total EDS score did not show an association with interest in psilocybin as a future treatment, suggesting that interest was not directly related to participants’ overall level of existential distress (**Table 2**). The average EDS score was relatively low at 16.13 ± 8.19 out of a maximum score of 50, indicating on average, participants experienced moderate levels of distress across the assessed items. Notably, a third of the respondents reported experiencing “great” or “unbearable” distress on at least one item. Specific areas of distress varied, with “being a burden to others” eliciting the highest reported distress and “having nothing to live for” causing the least distress. It is essential to acknowledge these specific areas of distress as they may have implications for the overall well-being and mental health of the participants.

The relatively low average EDS score could be attributed to various factors, including the participant population, survey semantics, failure to disclose, or survey fatigue. The lack of association between EDS scores and interest in psilocybin as a future treatment (p=0.507) could be attributed to a knowledge gap regarding the potential therapeutic uses of psilocybin. As 50% of participants selected “other” or wrote in “I don’t know,” this suggests a need for more comprehensive education for potential patients with literature on the therapeutic effects of psilocybin. Additionally, the options provided in the prompt may not have sufficiently covered the range of potential applications, leading to uncertainty and limited interest in psilocybin as a treatment option. Due to the sensitive nature of the EDS questions, such as “how distressed have you been by the thought that your life is meaningless,” participants may have felt discomfort with answering honestly in front of research team members that participants had no prior relationship with. Some participants may have experienced survey fatigue or a decreased level of interest toward the end of the survey, which took on average 13 minutes to complete, could explain the higher percentage of “I don’t know” responses. It is important to acknowledge this potential bias and consider ways to mitigate it in future studies, such as shortening the survey or incorporating breaks to maintain participant engagement throughout.

### Interest in psilocybin

Interest in psilocybin exhibited statistically significant associations with individual convictions about psilocybin’s therapeutic potential, concerns regarding safety risks, and its availability for medical, recreational, and spiritual purposes. Belief in PAT in treating stress or anxiety was significantly associated with interest in psilocybin as a treatment. Notably, while interest was statistically linked to the belief in the availability of psilocybin for medical (p=0.003), spiritual care (p=0.002), and recreational use (p=0.021), it was also significantly linked to worry about the safety risks of psilocybin (p=0.022) as seen in **Table 3**. Therefore, people who perceived the potential therapeutic benefits of psilocybin for stress and anxiety, or believed that it should be accessible for medical, spiritual, or recreational use, were more likely to express interest in receiving PAT.

**Table 3:** 2-sided chi-square hypothesis testing of interest in receiving psilocybin therapy by survey questions.

Demographic Information	Significance (2-sided)
Have You Ever Taken a Hallucinogen	1
Have You Ever Taken Medical Cannabis or “Marijuana”	1
Have You Ever Taken Recreational Cannabis or “Marijuana”	0.061
Have You Ever Taken a Medication for Depression, Anxiety, or Other Psychological Symptoms	0.802
I Am Knowledgeable About the Potential Clinical Use of Psilocybin	0.582
I Am Knowledgeable About the Risks and Side Effects of Psilocybin	0.437
I Know People That Have Used Psilocybin	0.155
I Am Worried About the Safety Risks of Psilocybin	0.022*^a^*
I Believe Psilocybin Can Treat Spiritual or Emotional Stress	0.005*^a^*
I Believe Psilocybin Should Be Accessible for Medical Use	0.003*^a^*
I Believe Psilocybin Should Be Accessible for Spiritual Use	0.002*^a^*
I Believe Psilocybin Should Be Accessible for Recreational Use	0.021
Have You Ever Seen Someone Under the Influence of Psilocybin	0.688
I Have Been Well Supported in my Spiritual and Emotional Care	0.216

The significance level for all tests is 0.050.

^a^: Significant finding.

Surprisingly, interest in psilocybin use was not significantly correlated with prior experience taking a hallucinogen, medical cannabis, nor recreational cannabis. Nor was interest in receiving therapy significantly associated with knowing other individuals that have used psilocybin previously or seeing someone under the influence of psilocybin. Furthermore, it is intriguing that interest correlated with the belief in psilocybin’s ability to address spiritual or emotional distress but not with a history of using medication for depression, anxiety, or other psychological disorder. Thus, personal experience or exposure to psilocybin, psychological medication, or recreational cannabis does not have to be a consideration for future implementation of psilocybin psychotherapy. Though, it is important to mention that many patients are reluctant to talk to providers regarding psychedelics and psychedelic use due to concerns about stigma and perceived lack of knowledge (20).

Survey participants’ interest was also not correlated with whether they feel well supported in their spiritual and emotional care (p=0.216). The range of data also indicates general satisfaction with current palliative care strategies, including chaplain services at the hospital. Consequently, it may be that the individuals surveyed may show little interest in alternative therapies despite believing in their effectiveness. Interestingly, there was no association with whether participants felt knowledgeable about risks, side effects, although there was a significant correlation with concern regarding safety risks of psilocybin.

### Concerns

Concerns regarding receiving therapeutic psilocybin in a clinical setting included psychosis (16.1%), lack of trained providers (16.1%), and exploitation by doctors/counselors (12.9%). However, these concerns were not significantly associated with interest in psilocybin as a future treatment. Participants reported personal experience, informal conversations, and movies/TV as common sources of knowledge on psilocybin. While experienced clinicians and academic research centers were identified as trusted sources, it is noteworthy that a significant portion of participants expressed uncertainty by selecting “I don’t know” when asked about the current data indicating the usefulness of psilocybin for specific conditions (**Figure 1**). This highlights the need for comprehensive education, dissemination of accurate information regarding psilocybin’s therapeutic potential, and implementing safety measures to address uncertainties and concerns. Interestingly, participants expressed lower concerns about stigma compared to financial costs and the scarcity of trained providers.

The lack of trained providers and concerns about potential exploitation by doctors and counselors are significant issues during clinical trials of PAT. There are unique ethical challenges faced by practitioners in PAT. The nature of these psychedelics, such as MDMA, psilocybin, and ketamine, can induce states of heightened emotional openness, introspection, and vulnerability, with potentially altered perceptions of touch, intimacy, and emotional connection (21). One paper explores potential risks of problematic interpersonal dynamics within ketamine-assisted psychotherapy, such as transference, grandiosity, unethical patient-provider relationships (22). The idea of grandiosity and transference is the underlying strong emotional attachments that can occur during psychedelic experiences, leading to idealized perceptions of the therapists that administer the drugs, or imbalanced power dynamics with unrealistic expectations of treatment (22). There is concern specifically within MDMA and psilocybin for their ability to induce states of “exchange of love,” can lead to potential therapist boundary transgressions and sexual abuse (23). Practitioners often recognize the importance of touch in psychedelic work, believing that it can be important catalyst to healing, but this leads to crucial conversations around the ethics of nonsexual, therapeutic touch for maintaining safety and boundaries in PAT (23). These challenges are particularly pertinent in the context of therapy with elderly patients or those nearing end of life with potential EXD, which could potentially increase their vulnerability to exploitation or abuse, as these hallucinogens can induce profound alterations in consciousness, emotional processing, and perception of self and reality.

A 2022 survey study of psychiatrists found that while the most common concerns were the lack of trained providers, the logistics of psychedelic-assisted therapy delivery, and the administration of psychedelics for patients with contraindications, the exploitation of patients was the least prevalent concern for psychiatrists (24). This shows a disconnect between patient and provider concern and a need for further provider education to understand patients’ concerns and safeguard against the potential for exploitation. This underscores the need for rigorous ethical guidelines and ongoing specialized training and supervision for practitioners. By maintaining clear boundaries, obtaining informed consent, and being mindful of the potential risks and benefits of therapeutic actions, PAT practitioners can prevent potential abuse and ensure that PAT is conducted in a safe and ethical manner to focus on the safety and wellbeing of patients, especially those nearing end of life.

Future research and interventions should also focus on enhancing comprehensive education by academic research centers and trained clinicians on the potential applications of psilocybin to address safety concerns and information about clinical use. Efforts should be made to address financial accessibility of treatment and ensure availability of trained professionals to reduce barriers to access. Recommendations also include adequate training for healthcare providers with safeguards against exploitation.

### Limitations

Improving the survey administration and participant well-being are crucial for future research. Shorter surveys that capture essential data are more likely to maintain participant engagement and minimize the chances of participant disinterest or agitation toward the end of the survey. To address potential discomfort or distress, consider placing potentially distressing inquiries towards the end of the survey. By placing potentially distressing inquiries at the end, participants may feel more present when completing prior sections regarding interest and psilocybin, which may be less emotionally challenging. The survey also asked primarily about diagnoses without prognosis, and future studies can look at the correlation between prognosis of disease to level of EXD experienced by the patient. Focusing on survey fatigue, the palliative care-specific patient experience, and concerns about safety and accessibility highlight areas for improvement in future studies. This survey was limited by small sample size, convenience sample, and non-experimental design.

### Future directions

To provide a more holistic experience for participants, we are considering integrating the survey into sessions facilitated by chaplains or mental health professional. Trained professionals can assist with navigating the survey’s emotional aspects, offer immediate support, and explore existential distress and deeper meaning. Alternatively, consider conducting group sessions or workshops where the survey is administered collectively followed by a facilitated discussion led by a chaplain or counselor. These formats allow for debriefing and shared experiences within a supportive group setting, serving as a platform to gain insights from others, find communal support, and identify shared coping strategies. Improving survey administration and participant well-being will contribute to a more participant-centered approach.

## Conclusion

Living with a non-curable illness can profoundly impact individual in social, emotional, physical, and spiritual aspects. EXD can arise from questioning life purpose, meaning, and existence, and is associated with anxiety about end-of-life and despair. Current medical approaches often fail to address the unique burden and distress experienced by patients with incurable illnesses. Psilocybin, a promising treatment for treatment-resistant depression, anxiety, addiction, and PTSD, has shown rapid and sustained symptom reduction in anxiety and depression for patients with life-threatening cancer. The integration of psilocybin and other psychedelics such as LSD and MDMA into the realm of palliative care holds great promise for alleviating the EXD and related burdens faced by individuals with incurable illnesses. These compounds offer a unique and rapid approach to symptom reduction, with lower risks and a potential for sustained therapeutic benefits, with social acceptability within groups of providers and the general population shifting towards support of such therapies.

This survey study explored attitudes towards PAT in a population of patients with incurable illnesses, providing insights into the potential interest and challenges of using PAT in this patient population in regard to EXD. Over half of the participants expressed interest and the belief in the potential therapeutic benefits of psilocybin for stress and anxiety was significantly associated with interest in its use. Concerns included risk of psychosis, risk of exploitation, and lack of trained providers. Patients’ awareness of concern for the risk of exploitation associated with PAT is an especially novel concern documented in this patient population. Overall, this highlighted the need for further education of risks and benefits or PAT by trained clinicians and rigorous training of clinicians with the establishment of safeguards against exploitation. Further research is needed to understand and implement this treatment approach, and to explore feasibility and acceptability regarding non-psilocybin psychedelics.

## Data availability statement

The raw data supporting the conclusions of this article will be made available by the authors, without undue reservation.

## Ethics statement

The study involving human participants were reviewed and granted exemption by Institution Review Board at the University of South Florida. The study was reviewed and approved by the Office of Clinical Research at Tampa General Hospital. Written and verbal informed consent to participate in this study was provided by the participants.

## Author contributions

JW: Writing – original draft, Visualization, Supervision, Project administration, Methodology, Investigation, Conceptualization. GM: Writing – original draft, Data curation. AM: Writing – original draft, Data curation. EC: Writing – original draft, Methodology, Formal analysis. SM: Writing – review & editing, Validation. RM: Writing – review & editing. DE: Writing – review & editing, Supervision, Project administration. KK: Writing – review & editing, Supervision.
